# Early postoperative clinical recovery of robotic arm-assisted vs. image-based navigated Total hip Arthroplasty

**DOI:** 10.1186/s12891-021-04162-3

**Published:** 2021-03-29

**Authors:** Nao Shibanuma, Kazunari Ishida, Tomoyuki Matsumoto, Koji Takayama, Yutaro Sanada, Masahiro Kurosaka, Ryosuke Kuroda, Shinya Hayashi

**Affiliations:** 1 Department of Orthopaedic Surgery, Kobe Kaisei Hospital, 3-11-15, Shinohara-Kita, Nada, Kobe, 657-0068 Japan; 2 Department of Orthopaedic Surgery, Kobe University Graduate School of Medicine, 7-5-1, Kusunoki, Chuo, Kobe, 650-0017 Japan; 3 Rehabilitation Center, Kobe Kaisei Hospital, 3-11-15, Shinohara-Kita, Nada, Kobe, 657-0068 Japan

**Keywords:** Robotic arm, Navigation, Early clinical outcome, Radiographic evaluation, Total hip arthroplasty

## Abstract

**Background:**

This study compared the early clinical recovery of total hip arthroplasty (THA) using computer navigation systems (nTHA) and robotic arm-assisted THA (rTHA).

**Methods:**

Thirty prospective subjects who underwent rTHA were clinically compared to 30 subjects who underwent nTHA. Clinical data (surgical time, intraoperative blood loss, pain severity, number of days to independent walking, and Harris Hip Score (HHS) at discharge), and radiographic parameters (inclination and anteversion angles) were statistically compared between the two groups.

**Results:**

Follow-up times were 24.3 ± 6.0 and 27.0 ± 7.0 days in the rTHA and nTHA groups, respectively. The surgical time (135.1 ± 13.9 min vs. 146.2 ± 12.8 min, *p* = 0.002), number of days to independent walking (7.2 ± 2.0 vs. 11.5 ± 3.0 days, *p* < 0.001), and postoperative pain using a numeric rating scale on postoperative days 7, 10,, and 14 (1.4 ± 0.9 vs. 2.2 ± 1.2, *p* = 0.005; 1.0 ± 0.8 vs. 1.8 ± 1.1, *p* = 0.002; 0.3 ± 0.5 vs. 1.1 ± 0.9, *p* < 0.001; respectively) were significantly reduced in the rTHA group compared to the nTHA group. The rTHA group showed a significantly higher postoperative HHS compared to the nTHA group (85.3 ± .3.2 vs. 81.0 ± 8.5, *p* = 0.014). No statistically significant difference was observed in radiographic parameters between the groups; however, the incidence of intraoperative target angle changes was significantly lower in the rTHA group than in the nTHA group (0/30 subjects [0%] vs. 11/30 subjects [36.7%], *p* < 0.001).

**Conclusion:**

The surgical time, postoperative pain, and number of days to independent walking were significantly shorter, and the HHS at discharge was significantly higher in the rTHA group than in the nTHA group. Thus, compared to the nTHA group, the rTHA group showed improved early clinical recovery.

## Background

Although total hip arthroplasty (THA) is a generally successful procedure [1], several factors, including patient demographics, surgical technique, and implant features, may affect short- and long-term outcomes [2–4]. One of the most important surgeon-controlled factors is component positioning [5]. Inadequate component positioning during THA may lead to an increased risk of postoperative complications such as bony/implant impingement, limited range of motion, dislocation, polyethylene wear, and loosening [6, 7]. Furthermore, malposition may also lead to leg-length discrepancy and muscle weakness due to a lack of offset, ultimately leading to patient dissatisfaction. Therefore, a precise and appropriate implant position is essential for good outcomes [6–8]. Several studies have assessed optimal acetabular cup alignment, with Lewinnek et al. defining the “safe zone” of cup alignment as 40° ± 10° inclination and 15° ± 10° anteversion [6]. However, consistently accurate component placement is difficult and high outliers from the “safe zone” are reported in 59–78% of conventional THA cases [6, 9, 10]. With the goal of achieving more accurate implant positioning during THA, computer-assisted orthopedic surgery (CAOS) has been developed [11], with good implant accuracy reported in THA using computer navigation systems (nTHA) [12, 13]. More recently, robotic arm-assisted THA (rTHA) has been introduced and is expected to enhance the accuracy of implantation. It has been shown to improve the accuracy of component positioning compared to manual THA [14]. Although both rTHA and nTHA are CAOS systems introduced to achieve more accurate implant positioning during THA, they have crucial differences. Navigation systems are typically passive systems that only provide patient-specific anatomical data with recommendations for bone resection and optimal implant positioning. The computer system does not actively control or restrain the motor function of the operating surgeon [15]. In contrast, rTHA uses computer software to convert anatomical information to a virtual patient-specific three-dimensional (3D) reconstruction of the pelvis that the operating surgeon uses to calculate and plan optimal implant positioning. An intraoperative robotic device helps to execute this preoperative patient-specific plan with a high level of accuracy. The reaming and acetabular component impaction are controlled by a haptic boundary while the surgeon physically moves the robot arm [11, 16, 17]. However, no studies have compared the clinical benefits, including surgical time and clinical outcomes, between rTHA and nTHA.

The purpose of this study was to compare early clinical recovery, including 1) surgical time, 2) days to independent walking, 3) intraoperative blood loss, 4) post-surgical pain severity, 5) the Harris Hip Score (HHS) at discharge, and 6) radiographic cup position between rTHA and nTHA. The primary outcomes were surgical time and early clinical outcomes such as postoperative pain, functional recovery to independent walking, and HHS at discharge. The secondary outcomes were intraoperative blood loss and radiographic cup position.

## Methods

### Subjects

This prospective comparative study was approved by the institutional review board of our institution, and informed consent was obtained from each patient. This study utilized data from a prospective total joint arthroplasty database containing demographic, clinical, and radiographic data on all primary THA procedures performed at our institution from February 2018 to January 2020. The clinical and radiographic results of a single senior surgeon at our institution following primary THA were reviewed. After robotic arm-assisted systems (Stryker Mako, Ft. Lauderdale, FL) were covered by insurance in Japan, the surgeon switched from nTHA (computed tomography [CT]-based hip navigation version 1.1, Stryker Navigation, Freiburg, Germany) to rTHA (Stryker Mako) for all primary THAs in June 2019. Among the 70 consecutive THA cases, female patients were selected to exclude the influence of sex on clinical outcome variance. Therefore, this study included 30 cases each of nTHA and rTHA.

### Surgical technique

All THAs were performed using the posterolateral approach, with patients in the lateral decubitus position. All hips were implanted with a cementless cup (Trident Acetabular Shell, Stryker Orthopedics, NJ, USA), a cemented stem (Exeter V40 Femoral Stem, Stryker Orthopedics, NJ, USA), ceramic 32-mm head (BIOLOX Delta V40 Ceramic Head, Stryker Orthopedics, NJ, USA), and non-elevated ultra-high-molecular-weight polyethylene liner (Trident X3 insert, Stryker Orthopaedics, NJ, USA). Preoperative CT was performed on all subjects for navigation and robotic arm-assisted systems. The slice thicknesses were 2.5 mm for navigation and 0.625 mm for robotic arm-assisted systems. The surgical procedures, including acetabular reaming and cup placement, differed between nTHA and rTHA, as described below.

### nTHA

After placement of a 4.0 mm tracker pin and acetabular exposure, surface mapping registration was performed to match the patients’ bony surface to the preoperative CT. Based on the instructions, the inner parts of the acetabulum were avoided for surface mapping. If a difference > 1.0 mm was found between surface mapping and the preoperative CT, re-registration was performed until the difference met the safety criteria. Thereafter, the surgeons performed acetabular reaming, during which the surgeons could see both anteversion and lateral inclination angles within the navigation monitor and control these angles manually. The navigation system could not visualize the reaming depth; therefore, step-by-step reaming of 1 mm was performed up to 1 mm smaller than the preoperative plan, from 6 mm smaller. The anterior, posterior, and medial wall thicknesses were checked in a timely manner to maintain an adequate reaming center and prevent wall collapse. After reaming, the surgeon placed the trial cup to check for adequate fit. Thereafter, the surgeon confirmed the anteversion and lateral inclination angle using navigation and placed the component manually until rigid fixation was achieved. Although the target inclination and anteversion angles were 40° and 20°, the components were placed in an adequate position to achieve better fixation when acceptable fixation was not achieved with the target angle. Two or three additional screws were inserted in all cases to achieve better stability. The final component angle, including anteversion and lateral inclination angle shown in navigation, were recorded.

### rTHA

After placement of a 4.0 mm tracker pin and acetabular exposure, surface mapping registration was performed to match the patients’ bony surface to the preoperative CT. During these procedures, the robotic systems were simultaneously set up by one of the assistants. Robotic systems require pointing within the inner parts of the acetabular space, as well as the extra-articular parts of the acetabulum. The difference between the registration and the preoperative CT was 0.5 mm in the rTHA. Single-step reaming using a reamer 1 mm smaller in size was performed with robotic arm assistance. This was followed by direct component placement without prior trial cup placement to check for adequate cup fit. The MAKO system guided the component placement angle and depth. If protrusions > 3 mm were found, same-size reaming was repeated to achieve an adequate depth of fixation. The target inclination and anteversion angles were 40° and 20°, respectively. Similar to the nTHA group, two or three screws were inserted in all cases.

The Mako Total Hip system does not track the stem itself during stem insertion. Thus, in all THAs (nTHA and rTHA), a cemented stem was inserted at the target angle (both varus/valgus and anteversion angles) in the femoral canal using a CT-based navigation system (NAV3i Platform; Stryker) namely, CT-Hip System V1.1 software (Stryker). The target of varus/valgus angles was 0°, and the anteversion was adjusted to match the anatomical neck anteversion.

The preoperative assessment, patient education program, and postoperative rehabilitation protocol were the same for both groups throughout the study period.

As a pain management protocol, 25 mL of 0.5% levobupivacaine hydrochloride was injected into the peri-articular portion soon after skin closure. From 1 day post-operation, 200 mg of etodolac was taken orally, twice a day for 14 days post-operation. A 25-mg diclofenac sodium suppository was used as a rescue medication.

### Evaluations

Patient demographics (age, sex, diagnosis, height, and weight) and clinical data (surgical time, intraoperative blood loss, pain severity, and the number of days to independent walking) were recorded for both groups. In addition, the Harris Hip Score (HHS) was determined preoperatively, 1 week before surgery, and at discharge. These data were statistically compared between the two groups.

### Pain severity

Post-surgical pain severity was evaluated using a numeric rating scale (NRS) for pain, with zero and ten indicating no and worst imaginable pain, respectively. The NRS during motion was evaluated on postoperative days (PODs) 1, 3, 7, 10, and 14.

### Physical function

The number of days to independent walking was defined as the period required for the patient to achieve a defined standard of independent walking using a T-cane. The following conditions had to be met with physical therapists observing the walking: walking for > 50 m with a T-cane, patient confidence in their ability to walk with the T-cane, and a timed up and go (TUG) result of < 13.5 s. The TUG was used to measure the time required to walk a 3.0 m distance, starting and ending with a sitting position.

To compare the accuracy of acetabular components, intraoperative and postoperative radiographic evaluations were performed. First, the incidence of intraoperative target angle changes to achieve component fixation was compared between the two groups. Over 5° difference from the target angles was defined as the intraoperative target angle change. Second, acetabular component placement, including version and inclination, was assessed radiographically using postoperative anteroposterior (AP) supine radiographs, as previously described [18]. Briefly, the software (Advanced Case Plan Ver 2.2; Stryker) created a horizontal reference line along the inferior aspect of the pelvic inter-ischial line as well as a complex of lines comprising a sphere, a concentric ellipse, and a bisecting line bisecting the ellipse along its long axis. While the lines comprising this complex could be manipulated individually, their relationships to each other remained unchanged. The sphere was then manipulated to fit the circumference of the acetabular cup, and the ellipse to fit the opening of the cup. The relative ratio of the axes of the ellipse corresponded to the cup version angle. The angle formed by the bisecting line and the inter-ischial reference line indicated the cup inclination angle.

This system could not differentiate between anteversion and retroversion. For version measurement, the cross-table lateral radiographs of all patients were reviewed using the Woo and Morrey [19] technique to ensure that they were anteverted. Based on the results for the inclination and anteversion angles, the incidence of outliers from the “safe zone” defined by Lewinnek et al. [6] were investigated. The radiographic measurements were performed by two observers blinded to the treatment, and the average used for assessment. The accuracy of the MAKO and navigation measurements was 0.1° and 0.1 mm and 0.5° and 1.0 mm, respectively. The accuracy of the radiographic measurements was 0.1°. The test-retest reliability of the measurements was excellent (interclass and intraclass correlation coefficients, 0.85–0.94).

### Statistical analysis

Data analyses were performed using a statistical software package (IBM SPSS Statistics for Windows, version 21.0, Armonk, NY, USA). Shapiro–Wilk tests were used to analyze normally distributed data. As the data were normally distributed, the data were expressed as mean ± standard deviation (SD). Comparisons between the two independent groups were performed using unpaired t-tests. The incidence of intraoperative target angle changes and the incidence of radiographic outliers from the safe zone were analyzed using Fisher’s exact test. A statistical a priori power analysis was performed using the G*Power software (version 3.1.9.2; Heinrich Heine Universität Düsseldorf, DE) to determine the sample size based on the differences between the days to independent walking of the two groups. A prespecified significance level of *α* < 0.05, a power level of 80%, and an effect size based on the results of the pilot study with ten cases (effect size *d* = 0.70) were used. The estimated sample size was 26 patients in each group, and the post hoc power analysis further confirmed that the power was 0.99. A *p-*value < 0.05 was set as the level of significance.

## Results

Patient demographic data are shown in Table 1. No significant differences were observed in age, body mass index, and preoperative TUG between the nTHA and rTHA groups. No acute intraoperative or postoperative complications were noted, including dislocation, infection, nerve palsies, or pin site-related complications such as fracture.

**Table 1:** Demographic data

	rTHA (*n* = 30)	nTHA (*n* = 30)	*p* value
Age (years old)	67.0 ± 7.6	64.8 ± 7.1	0.27
Height (cm)	154 ± 7	154 ± 4	
Weight (kg)	57.0± 9.4	56.2± 7.5	
Body Mass Index	23.9± 3.5	23.6± 2.7	0.7
sex	female	female	
Pre ope TUG (seconds)	9.7 ± 1.7	9.4 ± 2.3	0.27

*TUG* timed up and go test

The results for clinical outcomes are shown in Table 2. A significantly shorter surgical time was observed in the rTHA group than in the nTHA group. Intraoperative blood loss was comparable between the two groups. The number of days to independent walking was significantly shorter in the rTHA group than in the nTHA group. The NRS scores on PODs seven, ten, and fourteen were significantly lower in the rTHA group than in the nTHA group. Although no significant difference in preoperative HHS was observed between the rTHA and nTHA groups, rTHA showed a significantly higher postoperative HHS compared to the nTHA group.

**Table 2:** Clinical outcomes

		rTHA (*n* = 30)	nTHA (*n* = 30)	*p* value
Surgical time (min)		135.1 ± 13.9	146.2 ± 12.8	0.002*
Intraoperative blood loss (mL)		548.5 ± 203.9	568.7 ± 178.6	0.69
Days to independent walking (days)		7.2 ± 2.0	11.5 ± 3.0	< 0.001*
Pain (NRS)	POD 1	2.7 ± 1.2	3.0 ± 1.2	0.28
	POD 3	2.2 ± 1.1	2.6 ± 1.3	0.3
	POD 7	1.4 ± 0.9	2.2 ± 1.2	0.005*
	POD 10	1.0 ± 0.8	1.8 ± 1.1	0.002*
	POD 14	0.3 ± 0.5	1.1 ± 0.9	< 0.001*
HHS (points)	preoperative	44.1 ± 6.4	44.2 ± 5.2	0.81
	postoperative	85.3 ± 3.2	81.0 ± 8.5	0.01*

Values are presented as average ± standard deviation. *NRS* numeric rating scale, *POD* postoperative days, *HHS* Harris Hip Score

* Statistically significant difference

The results for acetabular component positions are shown in Table 3. The incidence of intraoperative target angle changes was significantly higher in the nTHA group than in the rTHA group. There were no statistically significant differences in the radiographic inclination and anteversion angles between the groups. The incidence of outliers from the safe zone was comparable between the two groups.

**Table 3:** Acetabular component position

		rTHA (*n* = 30)		nTHA (*n* = 30)		*p* value
Intraoperative target angle change; total		0/30 subjects (0%)		11/30 subjects (36.7%)		< 0.001*
Inclination	Anteversion	0/30 (0%)	0/30 (0%)	9/30 (30%)	4/30 (13.3%)	
Radiographic inclination (degrees)		42.2± 2.2 (38 - 47)		40.5± 4.5 (32 - 44)		0.07
Radiographic anteversion (degrees)		20.3± 1.6 (16 - 24)		19.9± 3.6 (14 - 33)		0.11
Outliers from the safe zone [6]		0/30 subjects (0%)		3/30 subjects (10%)		0.24

Values are presented as average ± standard deviation (range). * Statistically significant difference

## Discussion

The most important finding of the present study was that rTHA reduced surgical time and postoperative pain, and improved functional recovery to independent walking compared to nTHA. Furthermore, rTHA improved HHS at discharge compared to nTHA. These results suggest that rTHA is beneficial to postoperative early clinical recovery compared to nTHA.

A few studies have reported reduced pain, increased patient satisfaction, and improved functional outcomes as assessed using HHS and Forgotten Joint Score at a minimum 2 year follow-up in rTHA [20, 21]. These results compared the clinical outcomes between the rTHA and manual THA. To our knowledge, the present study is one of the few to compare the outcomes between rTHA and nTHA. The results showed that the rTHA reduced the postoperative numeric rating pain scale and postoperative functional recovery, although the radiographic outcomes are comparable to the nTHA. The short-term outcomes may be the metrics showing the major clinical differences between robotic and navigated systems, because of the very good outcomes of nTHA. Longer follow-up is still necessary to reveal the true clinical difference between the two.

The results showed a significantly shortened surgical time for rTHA compared to that for nTHA, although the surgical time in both groups was no longer short, if the time was compared to that ofthe manual THA. It should be noted that the relatively longer surgical time in both groups was influenced by several factors, including the use of a cemented stem with image-less navigation on the femoral side in this series. However, prolonged surgical time is well known as a risk factor for periprosthetic joint infection [22]; the results add to the merit of using rTHA compared to nTHA. There were however, no infection cases in both groups and the effort to shorten the surgical time should be continued further. As the surgical steps and implant types with the femur are consistent between the two groups, the effect is attributed to the difference in the surgical step on the acetabular side. The rTHA allowed for the use of a single reamer to prepare the acetabulum, followed by immediate component insertion compared to the nTHA technique. This typically involves sequential reaming of the target size with an assessment of bone preparation between each reaming stage [21], followed by the prior trial cup placement. It is considered that the reduction in reaming frequency and the omission of trial cup placement were the most likely reasons for the present results. One of the other possible explanations is registration method. Compared to the extra-articular surface mapping in the nTHA, both intra- and extra-articular surface mapping in rTHA enhance the accuracy to match the patients’ bony surface to the preoperative CT, thereby avoiding re-registration. Although the frequency of re-registration was not quantified, the advanced registration method in the rTHA might reduce the registration time. Conversely, setting up for the robotic systems did not increase the surgical time in this study. It is suggested that assigning the set up to the one of the assistants might not interfere with the surgical flow, leading to a longer surgical time. These possible influencing factors, including the registration methods and robot preparation, should be evaluated in future studies, as these are some of the limitations of this study.

In our study, no statistically significant differences were observed in radiographic inclination and anteversion angles, and the incidence of outliers from the safe zone between the techniques. These results were, however, underpowered. These results suggested that both navigation and robotics achieved equally acceptable component alignment. The inaccuracy of the actual cup position after THA is reportedly greatest in the mediolateral direction, in which the position is more lateral compared to the preoperative three-dimensional planning [23]. The study suggested that control of the mediolateral direction is difficult with manual reaming, and even with navigation. Although medialization was not evaluated in the present study, our technique of using a navigation system might result in insufficient medialization in some cases. In rTHA, acetabular reaming is controlled by the robotic device to ensure that the desired depth is reached for accurate restoration of the hip offset and center of rotation. One possible explanation for the superior results regarding postoperative pain and functional recovery is better medialization in rTHA than in nTHA. Further studies on the correlation between the amount of medialization and functional recovery are needed to reveal its true contribution. Furthermore, rTHA provides information for adequate depth and position during component impaction. Hasegawa et al. reported that 8.4% of cases experienced periprosthetic occult fractures of the acetabulum in primary THA, which might be ignored during surgery [24]. rTHA may reduce the rate of such occult fractures during impaction, which warrants investigation in future studies. The present results to investigate the incidence of intraoperative target angle changes found that the incidence is significantly higher in nTHA. The results suggested that the manual maneuver toward the target angle did not achieve hemisphere-shaped central reaming sufficient for initial rigid fixation of the acetabular component with the target angle. It is said that the insufficient press-fit fixation, which is influenced by the exactness of the reaming procedure, and the accurate cup insertion, significantly reduced acetabular component stability [25]. The present results, thus indicate that insufficient press-fit fixation and eccentric forces to the acetabulum might be increased in nTHA, leading to poor component stability, compared to rTHA. However, additional screw fixation might achieve acceptable stability in nTHA. One possible explanation for the reduced numeric rating scale for pain in rTHA is the better initial fixation stability via central reaming. It is considered that the earlier recovery to independent walking in the rTHA was caused by pain reduction.

The limitations of this study include the single-surgeon and single-institution study design. Since male patients were excluded, the results for male patients remain unknown; thus, further research is needed. The sample size was small, although the power analysis found that the sample size was sufficient for the primary outcomes. Some results, including the incidence of radiographic outliers from the safe zone, were underpowered. Future studies with larger sample sizes and prospective study designs could provide better evidence on robotic hip arthroplasty and build on the findings of this study.

The strength of this study is the homogeneity of the series in terms of technique and implants and the fact that all patients were operated on by the same surgeon.

## Conclusions

In summary, the surgical time was shorter; the NRS for postoperative pain on PODs 7, 10, and 14, the number of days to independent walking was significantly smaller, and the HHS at discharge was significantly higher in the rTHA group than in the nTHA group. Based on this series of results, rTHA improved early clinical recovery compared to nTHA.

## Data Availability

The datasets used and/or analyzed during the current study are available from the corresponding author on reasonable request.

## Abbreviations

3D: Three-dimensional
AP: Anteroposterior
CAOS: Computer-assisted orthopedic surgery
CT: Computed tomography
HHS: Harris Hip Score
NRS: Numeric rating scale
nTHA: Computer-navigated THA
POD: Postoperative day
rTHA: Robotic arm-assisted THA
SD: Standard deviation
THA: Total hip arthroplasty
TUG: Timed up and go

## References

[1] Knight SR, Aujla R, Biswas SP (2011). Total Hip Arthroplasty - over 100 years of operative history. Orthop Rev (Pavia).

[2] Cherian JJ, Jauregui JJ, Banerjee S, Pierce T, Mont MA (2015). What Host Factors Affect Aseptic Loosening After THA and TKA?. Clin Orthop Relat Res.

[3] Hug KT, Watters TS, Vail TP, Bolognesi MP (2013). The withdrawn ASR THA and hip resurfacing systems: how have our patients fared over 1 to 6 years?. Clin Orthop Relat Res.

[4] Janssen L, Wijnands KAP, Janssen D, Janssen M, Morrenhof JW (2018). Do Stem Design and Surgical Approach Influence Early Aseptic Loosening in Cementless THA?. Clin Orthop Relat Res.

[5] Callanan MC, Jarrett B, Bragdon CR, Zurakowski D, Rubash HE, Freiberg AA (2011). The John Charnley Award: risk factors for cup malpositioning: quality improvement through a joint registry at a tertiary hospital. Clin Orthop Relat Res.

[6] Lewinnek GE, Lewis JL, Tarr R, Compere CL, Zimmerman JR (1978). Dislocations after total hip-replacement arthroplasties. J Bone Joint Surg Am.

[7] Widmer KH, Zurfluh B (2004). Compliant positioning of total hip components for optimal range of motion. J Orthop Res.

[8] Dorr LD, Malik A, Dastane M, Wan Z (2009). Combined anteversion technique for total hip arthroplasty. Clin Orthop Relat Res.

[9] Steppacher SD, Kowal JH, Murphy SB (2011). Improving cup positioning using a mechanical navigation instrument. Clin Orthop Relat Res.

[10] Saxler G, Marx A, Vandevelde D, Langlotz U, Tannast M, Wiese M (2004). Cup placement in hip replacement surgery -- A comparison of free-hand and computer assisted cup placement in total hip arthroplasty -- a multi-center study. Z Orthop Ihre Grenzgeb.

[11] Sugano N (2013). Computer-assisted orthopaedic surgery and robotic surgery in total hip arthroplasty. Clin Orthop Surg.

[12] Iwana D, Nakamura N, Miki H, Kitada M, Hananouchi T, Sugano N (2013). Accuracy of angle and position of the cup using computed tomography-based navigation systems in total hip arthroplasty. Comput Aided Surg.

[13] Sugano N, Takao M, Sakai T, Nishii T, Miki H (2012). Does CT-based navigation improve the long-term survival in ceramic-on-ceramic THA?. Clin Orthop Relat Res.

[14] Domb BG, Chen JW, Lall AC, Perets I, Maldonado DR. Minimum 5-Year Outcomes of Robotic-assisted Primary Total Hip Arthroplasty With a Nested Comparison Against Manual Primary Total Hip Arthroplasty: A Propensity Score-Matched Study. J Am Acad Orthop Surg. https://10.5435/JAAOS-D-19-00328. 2020. 10.5435/JAAOS-D-19-00328 32109923

[15] Parratte S, Argenson JN, Flecher X, Aubaniac JM (2007). Computer-assisted surgery for acetabular cup positioning in total hip arthroplasty: comparative prospective randomized study. Rev Chir Orthop Reparatrice Appar Mot.

[16] El Bitar YF, Jackson TJ, Lindner D, Botser IB, Stake CE, Domb BG (2015). Predictive value of robotic-assisted total hip arthroplasty. Orthopedics.

[17] Nawabi DH, Conditt MA, Ranawat AS, Dunbar NJ, Jones J, Banks S (2013). Haptically guided robotic technology in total hip arthroplasty: a cadaveric investigation. Proc Inst Mech Eng H.

[18] Domb BG, Redmond JM, Louis SS, Alden KJ, Daley RJ, LaReau JM (2015). Accuracy of Component Positioning in 1980 Total Hip Arthroplasties: A Comparative Analysis by Surgical Technique and Mode of Guidance. J Arthroplast.

[19] Woo RY, Morrey BF (1982). Dislocations after total hip arthroplasty. J Bone Joint Surg Am.

[20] Perets I, Walsh JP, Close MR, Mu BH, Yuen LC, Domb BG (2018). Robot-assisted total hip arthroplasty: Clinical outcomes and complication rate. Int J Med Robot.

[21] Bukowski BR, Anderson P, Khlopas A, Chughtai M, Mont MA, Illgen RL (2016). Improved functional outcomes with robotic compared with manual Total hip Arthroplasty. Surg Technol Int.

[22] Cordero-Ampuero J, de Dios M (2010). What are the risk factors for infection in hemiarthroplasties and total hip arthroplasties?. Clin Orthop Relat Res.

[23] Hassani H, Cherix S, Ek ET, Rudiger HA (2014). Comparisons of preoperative three-dimensional planning and surgical reconstruction in primary cementless total hip arthroplasty. J Arthroplast.

[24] Hasegawa K, Kabata T, Kajino Y, Inoue D, Tsuchiya H (2017). Periprosthetic Occult Fractures of the Acetabulum Occur Frequently During Primary THA. Clin Orthop Relat Res.

[25] Tabata T, Kaku N, Hara K, Tsumura H (2015). Initial stability of cementless acetabular cups: press-fit and screw fixation interaction--an in vitro biomechanical study. Eur J Orthop Surg Traumatol.

